# Crystal Structure of Bfr A from *Mycobacterium tuberculosis*: Incorporation of Selenomethionine Results in Cleavage and Demetallation of Haem

**DOI:** 10.1371/journal.pone.0008028

**Published:** 2009-11-25

**Authors:** Vibha Gupta, Rakesh K. Gupta, Garima Khare, Dinakar M. Salunke, Anil K. Tyagi

**Affiliations:** 1 Department of Biochemistry, University of Delhi South Campus, New Delhi, India; 2 Ram Lal Anand College, University of Delhi, New Delhi, India; 3 National Institute of Immunology, New Delhi, India; University of California Berkeley/JGI, United States of America

## Abstract

Emergence of tuberculosis as a global health threat has necessitated an urgent search for new antitubercular drugs entailing determination of 3-dimensional structures of a large number of mycobacterial proteins for structure-based drug design. The essential requirement of ferritins/bacterioferritins (proteins involved in iron storage and homeostasis) for the survival of several prokaryotic pathogens makes these proteins very attractive targets for structure determination and inhibitor design. Bacterioferritins (Bfrs) differ from ferritins in that they have additional noncovalently bound haem groups. The physiological role of haem in Bfrs is not very clear but studies indicate that the haem group is involved in mediating release of iron from Bfr by facilitating reduction of the iron core. To further enhance our understanding, we have determined the crystal structure of the selenomethionyl analog of bacterioferritin A (SeMet-BfrA) from *Mycobacterium tuberculosis* (Mtb). Unexpectedly, electron density observed in the crystals of SeMet-BfrA analogous to haem location in bacterioferritins, shows a demetallated and degraded product of haem. This unanticipated observation is a consequence of the altered spatial electronic environment around the axial ligands of haem (in lieu of Met52 modification to SeMet52). Furthermore, the structure of Mtb SeMet-BfrA displays a possible lost protein interaction with haem propionates due to formation of a salt bridge between Arg53-Glu57, which appears to be unique to Mtb BfrA, resulting in slight modulation of haem binding pocket in this organism. The crystal structure of Mtb SeMet-BfrA provides novel leads to physiological function of haem in Bfrs. If validated as a drug target, it may also serve as a scaffold for designing specific inhibitors. In addition, this study provides evidence against the general belief that a selenium derivative of a protein represents its true physiological native structure.

## Introduction

Despite more than a century of research on various aspects of tuberculosis (TB), control of this disease caused by *Mycobacterium tuberculosis* (Mtb), has been difficult due to complex and nature of the host-pathogen interactions, which poses a challenge for global public health. A serious limitation of the current TB treatment is its inability to completely eliminate this persisting pathogen from the host [1], [2]. The problems of the disease are being amplified by a recent surge of multidrug–resistant cases and accepted synergy between TB and AIDS epidemics [3]. This has necessitated an urgent search for new antitubercular drugs against novel targets and combined efforts by several organizations are presently under way to determine the three dimensional structures of a large number of mycobacterial proteins for structure-based drug designing. Iron is required for the growth of tubercle bacilli in broth culture as well as in macrophages and thus represents a crucial requirement for infection by this pathogen [4], [5]. Due to its two readily interchangeable oxidation states (II) and (III), iron is an extremely useful redox mediator in biology. It is an indispensable cofactor for proteins participating in critical cellular processes such as electron transfer, oxygen transport, DNA synthesis, nitrogen fixation and for production of haemoproteins. Though iron is essential, the excess of free iron is potentially toxic as it catalyzes the production of reactive oxygen by Haber-Weiss/Fenton reactions, which cause oxidative damage to the cell. Thus, the cellular levels of iron have to be tightly regulated, for which efficient iron acquisition and storage mechanisms have been developed by all living organisms [6]. Safe iron storage, detoxification and appropriate delivery of iron for biosynthetic functions in a cell are carried out by a superfamily of proteins known as ferritins that are widely found in all domains of life. A subfamily of ferritin superfamily found in eubacteria, called bacterioferritins (Bfrs), differ from ferritins in having additional noncovalently bound haem groups. The haem moiety in the Bfrs is normally in the form of protoporphyrin IX, though Bfr of *Desulfovibrio desulfuricans* uses iron-coproporphyrin III type of haem [7]. Although it has been suggested that haem may be needed for iron extraction from the Bfr structure, the exact function of this moiety remains largely unknown [8], [9]. Studies relating the role(s) of bacterioferritins in the physiology of the cell from many bacterial species present a diverse picture. Depending on the bacterial species, bacterioferritins have been shown to store excess iron for the protection of bacterial cells against iron overload and/or against oxidative stress as well as to act as a source of the metal during iron starvation and as virulence factors [10].

The Mtb genome reveals the presence of two putative iron-storage proteins, namely, BfrA (Rv1876)–a bacterioferritin and BfrB (Rv3841)–a ferritin like protein [11]. It is expected that the expression of these genes would be upregulated in high-iron conditions and reduced in low-iron conditions as has been shown in other bacteria [12], [13]. As anticipated, the transcription of *bfrB* has been found to be repressed *in vitro* under iron-limited conditions [14]. Interestingly, *bfrA* in Mtb is controlled by three promoters, of which two are repressed by iron, whereas, the third is activated by high levels of iron [15]. Therefore, intriguingly, mRNA of *bfrA* gene in Mtb is produced under both low- and high-iron conditions, thus suggesting that BfrA may have an additional role than storage of iron *in vivo*. It is quite possible that the mRNA pool of this gene has to be always available so that under iron overload conditions the gene for the storage of toxic iron can be translated quickly. The firmly regulated expression of BfrA appears to be crucial for the adaptation and survival of tubercle bacilli in the host. Hence, it represents a promising target for structure determination. We have determined the crystal structure of selenomethionyl analog of bacterioferritin A (SeMet-BfrA) from *M. tuberculosis* and carried out in-depth analysis to identify sub regions of structure unique to *Mycobacterium* species. These hot spots distinguish Mtb BfrA from its peers and can be exploited for specific inhibitor design against the species. We also discuss a possible route for the uptake/release of iron into Mtb BfrA. Compounds targeting this route could inhibit the iron storage function of the protein and interfere with bacterial iron metabolism proving extremely valuable for chemotherapy of the disease.

Selenium is by far the most commonly used anomalous scatterer for MAD studies and hence engineering of selenomethionyl (SeMet) proteins for structure determination is now routine. Substituting SeMet for methionine usually has no adverse effects on the labeled protein, although change in kinetic properties after SeMet labeling of phosphomannose isomerase has been reported [16]. The crystal structure of SeMet-BfrA additionally demonstrates that SeMet incorporation changes the electronic environment in the haem-binding pocket of the protein leading to demetallation and cleavage of haem ligand.

## Results

### Purification and Spectral Features of Mtb SeMet-BfrA

**Figure 1:**
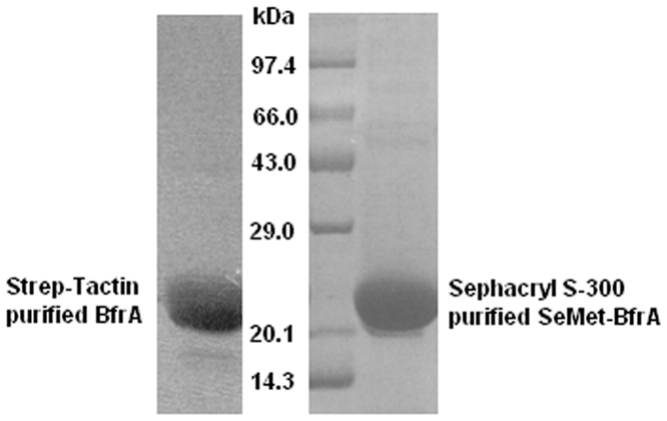
Purity of recombinant Mtb BfrA. 12.5% SDS-PAGE analysis of Strep-Tactin purified native BfrA vs Sephacryl S-300 purified SeMet-BfrA.

**Figure 2:**
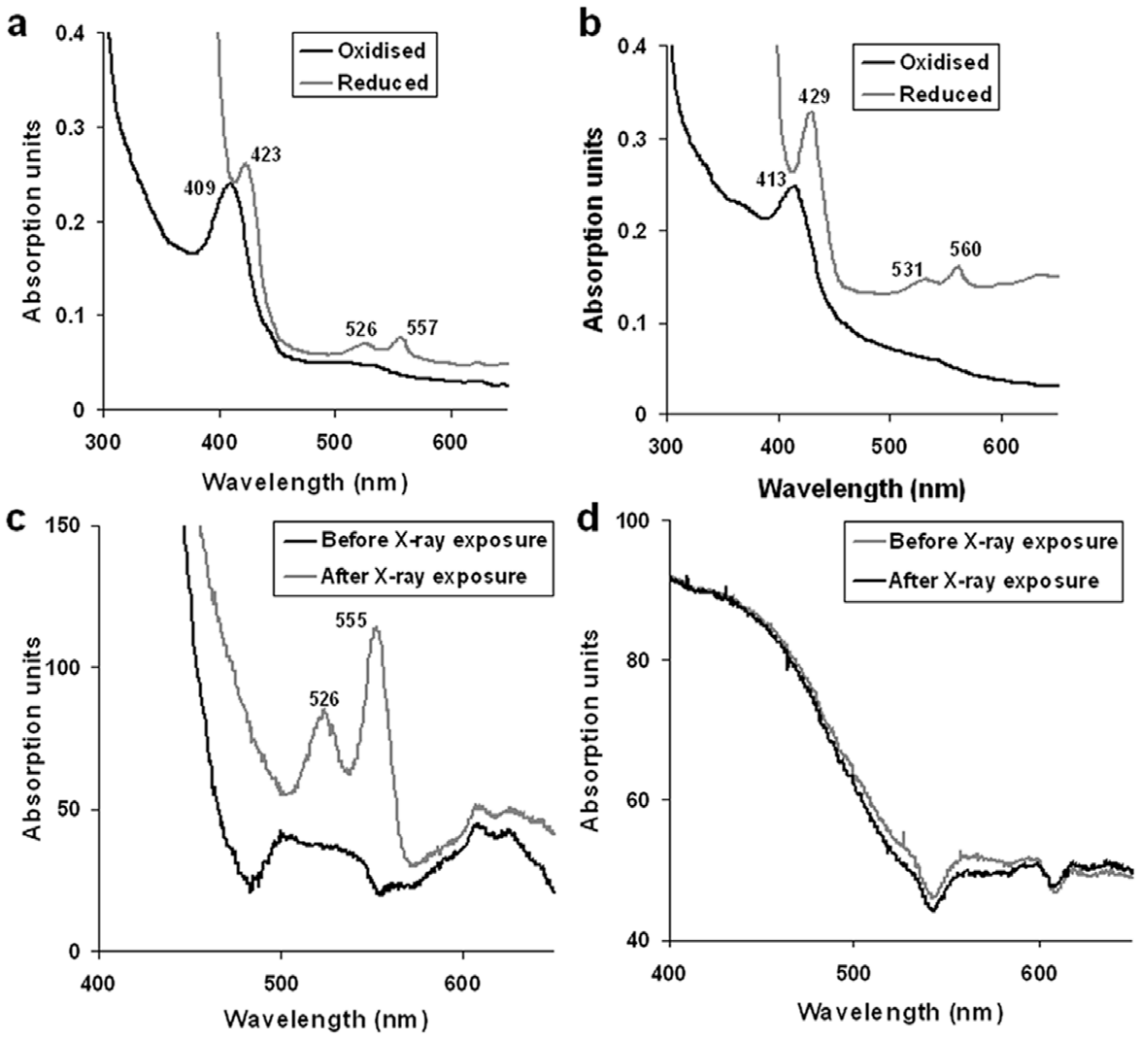
Spectral studies of Mtb native and SeMet BfrAs in solution and crystalline form. U.V.-visible spectra of (**a**) Native and (**b**) SeMet BfrA in oxidized (black) and reduced (gray) states. Single crystal microspectrophotometry at 100 K on crystals of (**c**) Native and (**d**) SeMet BfrA, before (black) and after (gray) X-ray exposure.

Expression of SeMet-BfrA is much low compared to the native BfrA as reported earlier [17] and hence necessitated changes in the purification procedure. The purity of SeMet analog with changed protocol was comparable to affinity purified BfrA (Figure 1). The absorption spectra in solution for both native and SeMet derivatized BfrA are shown in Figure 2a & 2b, respectively and exhibit similar profiles. A prominent Soret band at 409 nm for the native and 413 nm for the SeMet protein indicates the presence of oxidized haem. Reduction with sodium dithionite shifts the Soret band to 423 nm for native BfrA and to 429 nm for SeMet-BfrA. The α and β bands are also detectable with respective maxima at 557 nm and 526 nm for the native protein and 560 nm and 531 nm for the SeMet derivative. The substitution of sulfur by selenium is known to result in consistent small red shift (∼4 nm) in the visible spectrum [18], as is evident from the visible spectroscopic data obtained from the two proteins. The broad background in the visible region (∼600 nm) for the reduced SeMet-BfrA may reflect heterogeneity in the local environment of haem iron. To further explore the scenario vis-à-vis the presence of BfrA haem in the solid phase and possible effect of X-radiation, single crystal microspectroscopy was performed on native and SeMet-BfrA crystals before and after exposure to the X-ray beam (Figure 2c & 2d). Exposure of native Mtb BfrA crystal to the X-ray (gray curve in Figure 2c) caused appearance of sharp α and β bands at 555 nm and 526 nm, respectively, akin to chemical reduction of the protein in solution (Figure 2a), suggesting that in the crystalline form the haem of Mtb BfrA was in the oxidized state before irradiation (black curve in Figure 2c) but got reduced by exposure to the X-rays. Crystals formed from Mtb SeMet-BfrA display a featureless optical absorption spectrum before as well as after the X-ray exposure (Figure 2d). No visible reduced peaks can be assigned for α and β bands even on higher exposure to the X-rays. The most plausible explanation for this observation originates out of the crystal structure of SeMet-BfrA that reveals the presence of a degraded haem moiety and thus provides clarification for the absence of haem associated signatures (vis-à-vis α and β bands) during microspectroscopy of SeMet-BfrA crystals. Furthermore, mass spectrometry of native and SeMet-BfrA crystals does not show any haem peak (616Da) in the latter sample as opposed to the former, thus, providing an additional evidence for the degradation of haem moiety in the SeMet-BfrA crystals (Figure S1).

### Crystal Contents and Structure Quality

The crystal structure of Mtb SeMet-BfrA has been determined by Molecular Replacement (MR) using the structure of the recently published *M. smegmatis* BfrA as template [19] and refined to a final R_work_ value of 19% and an R_free_ value of 23% at 2.5 Å resolution. The asymmetric unit contains six copies of the protein (159 residues of BfrA and 2 linker residues), three demetallated and degraded haem molecules (labeled unknown ligand [UNL] in PDB 2wtl), twelve iron atoms and 170 water molecules. Except for a couple of residues at the N- terminus that were not included in the model, the electron density was generally well defined along the protein chain. Stereochemical analysis of the final model using the program PROCHECK [20] shows a good stereochemistry, with 97.1% of the residues in favored, 2.8% of the residues in generously allowed and 0.1% of residues in disallowed regions of Ramachandran plot.

### Structure of Mtb SeMet-BfrA

**Figure 3:**
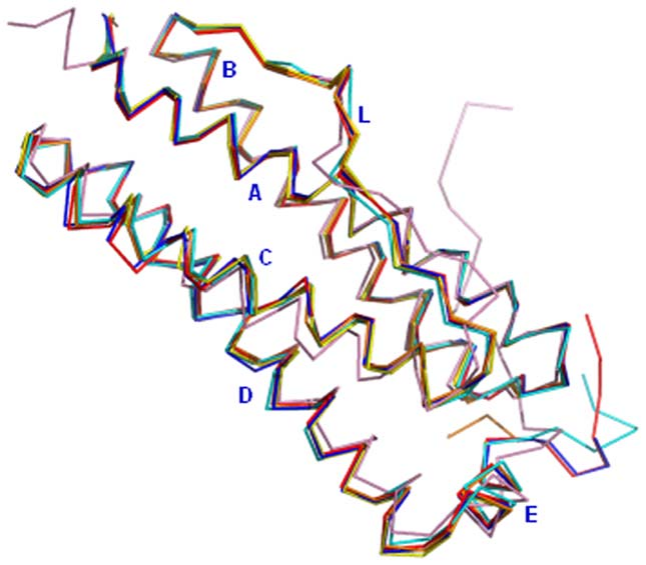
Conserved architecture of Mtb BfrA. Superposition of Mtb SeMet-BfrA (Chain D, shown in blue) with monomers of bacterioferritins from other prokaryotes. The PDB ID followed by the Chain ID used for superposition and the color of the monomer is given in parenthesis. *M. smegmatis* (3bkn∶A; red), *E. coli* (2htn∶H; orange), *A. vinelandii* (1sof∶E; yellow), *R. capsulatus* (1jgc∶A; cyan) and *D. desulfuricans* ((1nf6∶M; pink). Also the five helices (A–E) and the L-loop are labeled in blue.

The structure of Mtb SeMet-BfrA exhibits the highly conserved architecture of ferritin superfamily where the complete biological molecule is assembled into an almost spherical shell by the symmetrical association of 24 equivalent monomers that are related by operation of 4-, 3- and 2-fold symmetry axes (Figure 3). The protein shell created by subunit packing encloses a large cavity of ∼80 Å that occupies approximately 30% of the total macromolecular volume. The root mean square deviation (RMSD) between equivalent Cα atoms after global superimposition of Mtb SeMet-BfrA on *M. smegmatis* (3bkn∶chainA), *Escherichia coli* (2htn∶chainH), *Azotobacter vinelandii* (1sof∶chainE), *Rhodobacter capsulatus* (1jgc∶chainA) and *D. desulfuricans* (1nf6∶chainM) BfrA is 0.29 Å, 0.52 Å, 0.53 Å, 0.64 Å and 1.07 Å, respectively. As expected the structure of Mtb BfrA is closest to its homologue from saprophytic mycobacterial species *M. smegmatis*. Each subunit is composed of four long helices (namely, A from Pro5-Trp35, B from Thr38-Leu65, C from Leu83-Lys111 and D from Thr114-Leu144), a fifth short helix (E from Glu146-Cys153) at the C-terminus and a long extended L-loop (from Asp66-Thr82) that connects helix B to C (Figure 3).

### Haem-Binding Pocket

**Figure 4:**
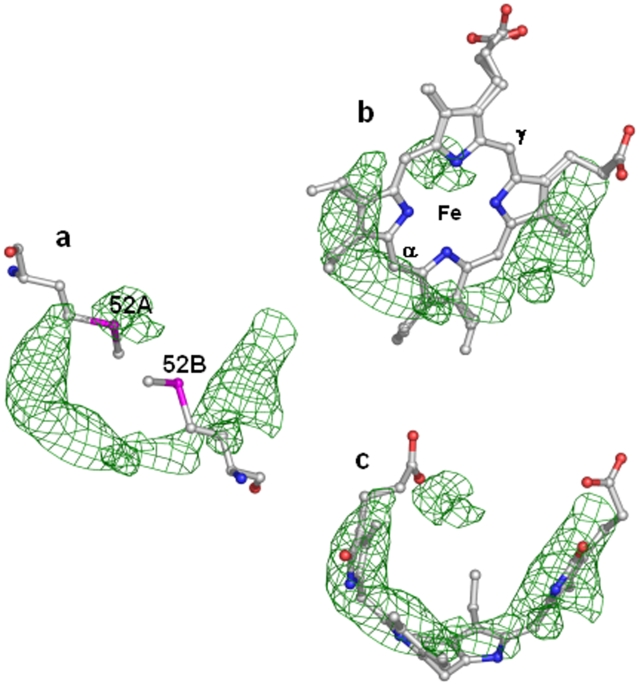
The *F_o_−F_c_* electron density map in the haem-binding region of Mtb SeMet-BfrA. (**a**) Sigma weighed *F_o_−F_c_* (green mesh contoured at 2.3σ) map around the haem axial ligands SeMet52 from two subunits (shown as sticks in atom type colors) without any ligand build in it. (**b**) Superimposition of the haem coordinates from *M. smegmatis* Bfr (3bkn) on the *F_o_−F_c_* map demonstrates haem degradation at γ position. The α and γ methene bridges of the haem moiety are marked in the figure. (**c**) Similar superposition of the biliverdin IX-γ (coordinates obtained from HIC-Up) illustrates partial fit to the density. Haem/biliverdin are modeled as sticks in atom type colors.

In the Mtb SeMet-BfrA structure, this haem-binding pocket is lined with residues Leu19, Ile22, Asn23, Phe26, Arg45, Phe49, SeMet52, Arg53, Ala55, Glu56, Tyr71 and the equivalent twofold-axis-related residues. Initially, in either the 2*F*_o_−*F*_c_ or *F*_o_−*F*_c_ electron density maps, without haem being kept in the phasing model, significant continuous positive density appeared corresponding to the location where haem is positioned in Bfrs from other organisms (Figure 4a). This density lies between the side chains of two SeMet52 residues belonging to two monomers related by the twofold axis. Attempts to build haem with either full or low occupancy only resulted in high temperature factors and additionally showed negative difference electron density at metal position indicating its absence in the porphyrin moiety. Moreover, the circumference of the horse-shoe shaped electron density ring appearing at the documented haem position in the SeMet-BfrA structure was larger than expected for the haem porphyrin ring and appeared broken between two five-membered pyrroles (Figure 4b) strongly suggesting the presence of a metal-free and degraded porphyrin derivative. On further exploring the shape of the electron density, the break in the density advocated that the porphyrin ring is cleaved at its γ-meso carbon position, resulting probabaly in the generation of biliverdin IX-γ isomer as the main end product. At first glance the density appeared to fit biliverdin IX-γ (Figure 4c) but further refinement of this ligand with its standard stereochemical library exhibited severe distortion in the planarity of the pyrroles. Moreover, since the mass spectrometry analysis of Mtb native and SeMet-BfrA crystals was unable to provide a clear evidence about the presence of biliverdin as the haem degradation product (Figure S1), this ligand has been designated as ‘unknown ligand’ (UNL) in the deposited PDB.

**Figure 5:**
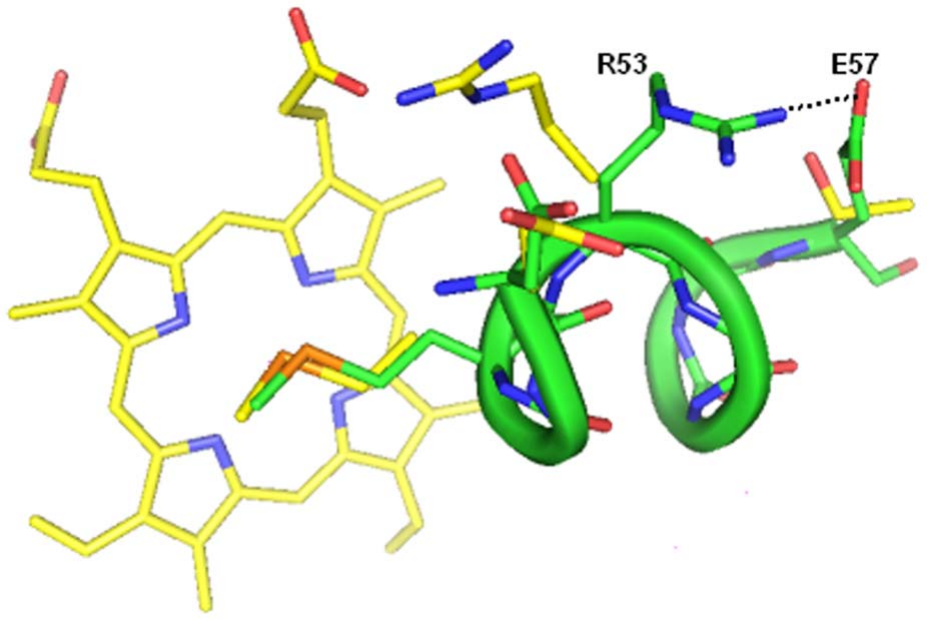
Comparative view of haem binding pocket of Mtb and *M. smegmatis* BfrA. The region 50–57 from Mtb SeMet-BfrA (protein chain shown as green tube and the amino acid side chains depicted as sticks) is superimposed with the same region including haem moiety (shown as sticks) from *M. smegmatis* (yellow) to highlight the flipped orientation of R53 in Mtb and loss of an interaction with haem. The flipped conformation that coincides with formation of a salt bridge between R53 and E57 (T57 in *M.smegmatis*) is shown with a black dashed line.

Comparison of haem binding pocket from Mtb SeMet-BfrA and *M. smegmatis* BfrA structures reveals subtle differences in this region (Figure 5). The Arg53 in Mtb BfrA has flipped its orientation by formation of a salt bridge with Glu57, which in *M. smegmatis* is mutated to Thr57. This altered orientation of Arg53 that results in the loss of an interaction with haem propionate maybe a chance factor or could have physiological relevance (discussed later).

### The Ferroxidase Centre

**Figure 6:**
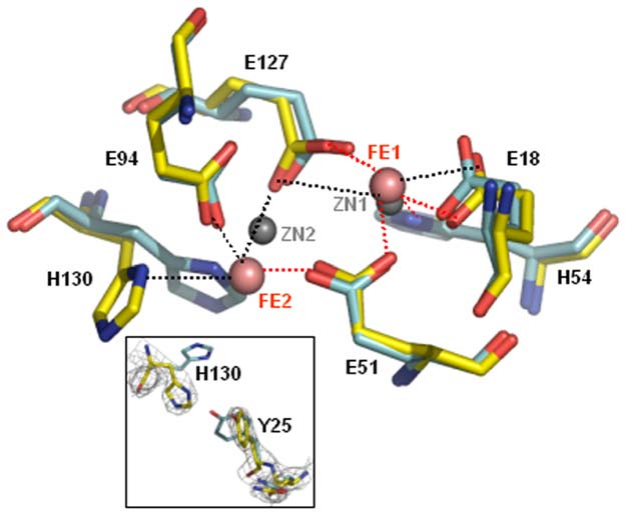
Ferroxidase centre of Mtb SeMet-BfrA. Ferroxidase centre of Mtb SeMet-BfrA (yellow) showing the metal coordinating residues is superposed with that of *M. smegmatis* BfrA (PDB: 3bkn; gray). The iron atoms in Mtb are shown as brown spheres, whereas the corresponding zinc metal ions in the homologue are shown as gray spheres. Red dashed lines indicate bonds, while black dashed lines indicate distances greater than 2.6 Å. The inset shows the electron density in a 2*Fo−Fc* map contoured at 1.8σ around H130 and Y25 (gray mesh) exhibiting its alternate conformation in the structure of Mtb SeMet-BfrA as against the same region from *M. smegmatis* structure.

The initial step in the process of iron uptake by (bacterio)ferritins involves oxidation of ferrous iron by molecular oxygen through a binuclear di-iron centre, the ferroxidase centre [21], [22]. Typically, the two iron ions comprising the di-iron centre are stabilized by glutamate and histidine residues and are mostly conserved in the Bfr family. The residues forming the symmetric ferroxidase centre in Mtb SeMet-BfrA are: Glu18, Glu51, Glu94, Glu127, His54 and His130. Glu51 and Glu127 are the bridging ligands whereas the rest of the residues are the capping ligands to the two metal ions (labeled Fe1 and Fe2 in Figure 6). In the ferroxidase centre of Mtb SeMet-BfrA, initially based on the electron density, full occupancy was assigned for both the iron atoms, but temperature factors were found to be high and density also did not compare with scattering expected from an iron atom. Conversely, AAS or ICP-MS of protein sample did not show any metal other than iron and selenium (data not shown), therefore, occupancy was slowly reduced so as to constrain the temperature factors of the two iron atoms equal to that of the protein molecule. The best temperature factors and R_free_ value were obtained when iron was refined with 0.3 occupancy in both Fe1 and Fe2 sites. Di-iron site interatomic distances involving the two metal ions and protein residues are listed in Table 1.

**Table 1:** Di-iron Site Interatomic Distances^a^.

ligand	Fe1	Fe2
E^18^OE1	2.9 (2.6–3.1)	
E^18^OE2	2.8 (2.6–2.9)	
H^54^ND1	3.1 (2.7–3.7)	
E^51^OE1	2.2 (1.9–2.5)	
E^51^OE2	3.4 (3.0–4.0)	2.5 (2.3–3.0)
E^127^OE1	3.4 (3.1–3.8)	2.6 (2.1–3.1)
E^127^OE2	2.3 (1.9–2.5)	3.9 (3.4–4.4)
H^130^ND1		3.6 (3.1–4.6)
E^94^OE2		2.9 (2.3–3.2)

a The average distance (Å) is reported for all six subunits in the asymmetric unit, with ranges in parentheses.

### Three-Fold Channels, Four-Fold Channels and the B-Site

**Figure 7:**
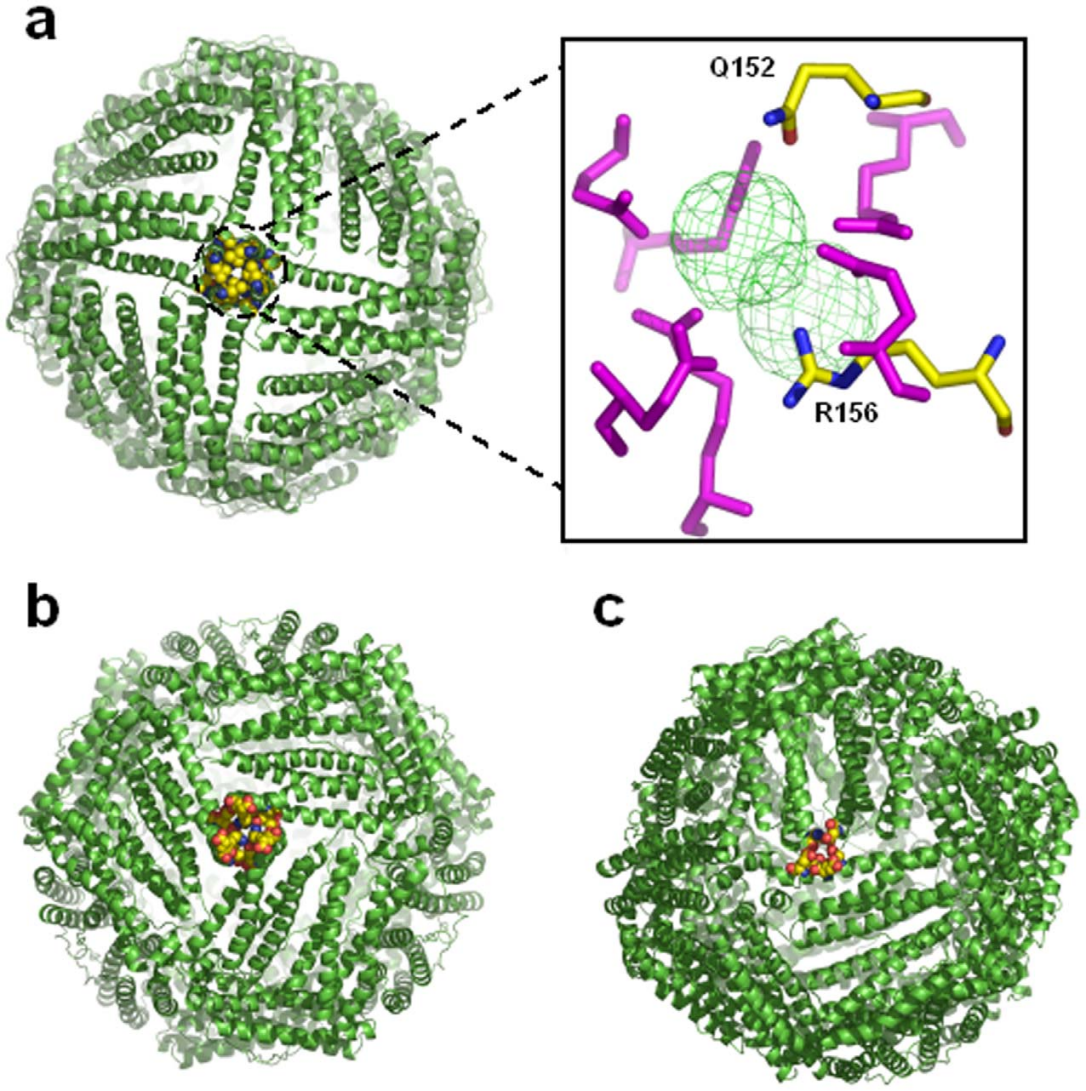
Channels and pores of Mtb SeMet-BfrA. Molecular representation of the (**a**) four-fold channel, (**b**) three-fold channel and (**c**) the B-pore in the Mtb SeMet-BfrA protein. The amino acids that line the channel or the pore are shown in the CPK representation and the remaining portions are shown in the cartoon representation. The two spherical electron density peaks (in a *F_o_−F_c_* difference map contoured at 5.0σ) of an unidentified ligand at the four-fold axis in the structure of Mtb SeMet-BfrA are shown as inset to figure 7a. The residues Q152 and R156 are shown as sticks colored by atom type from one monomer whereas symmetry related residues at the 4-fold are colored in magenta.

Despite a very similar overall topology, a remarkable heterogeneity is observed for the subunits of different classes of ferritin superfamily and is the reason for different possible functions executed by its various members [10]. Solvent channels traversing the external surface of the assembled holobacterioferritin/ferritin macromolecule to the inner cavity provide a pathway for iron and electrons/protons controlling the redox potential and the pH, and influencing the nature of the mineral formed in the core [23]. In Mtb SeMet-BfrA, the four-fold channel is lined with Leu148, Gln152, Val154 and Arg156 (Figure 7a) and appears mostly nonpolar. This seems to be unfavorable for Fe^2+^ ion entrance but definitely holds some other unidentified ligand in the crystal structure. The two strong spherical electron density peaks (in the *F*_o_−*F*_c_ difference map) placed in tandem down the four-fold axis are unambiguous and entrapped by Arg156 and Gln152 from all four subunits. This suggests passage of some ligand (nonpolar or anionic) through this channel (Figure 7a inset). We tried modeling the usual anions (specially chloride ion as the protein is crystallized with NaCl) but the strong difference electron density (>5σ) could not be accounted for and hence, we have designated this density as unknown ion (UNX).

The three-fold channel lined with hydrophilic residues Arg109, Glu110, Thr114, Val118, Glu121 and Lys122 (Figure 7b) is a mix of negative and positive charges that may create an electrostatic field to direct ferrous ions into the interior of the protein shell similar to that proposed for recombinant human H-chain ferritin [24]. A pore, termed the B-site [7], is large enough to accommodate an iron atom and is lined mostly with negatively charged residues Asn34, Asp66, Asp132, Glu135, Thr136 and Glu139 (Figure 7c) and hence maybe the most likely route for iron entry/exit to/from the Bfr cavity.

## Discussion

### Haem Degradation

Haems or iron-porphyrin complexes are prosthetic groups involved in many essential biological processes such as oxygen transport, respiration, photosynthesis, drug detoxification and signal transduction [25]. However, free haem can display both protective [26], [27], [28] and deleterious activities [29], [30]. For these reasons, haem homeostasis is strictly regulated. The main catabolic fate of haem *in vivo* is its enzymatic degradation by haem oxygenase (HO) which regiospecifically oxidizes the α-meso position of haem to form the α-biliverdin isomer, carbon monoxide and free iron [31]. Furthermore, it is documented that when haem iron undergoes redox reactions in the presence of ROS, nonenzymatic haem degradation can occur. Unlike enzymatic degradation, which specifically attacks the α-methene bridge, reactive oxygen species randomly attack all the carbon methene bridges of the tetrapyrrole rings, producing all four possible α, β, γ, δ-biliverdin isomers and releasing free iron [32]. Our study, regardless of the precise nature of the product, clearly identifies the haem degradation phenomenon occurring at the γ-methene bridge (Figure 4), indicating a non-enzymatic cleavage of haem. The regiochemistry of haem oxidation by mammalian HO is strongly governed by the orientation of the haem in the active site cavity, and this orientation is largely determined by ionic interactions of haem propionates with the protein residues lining the cavity [33]. In spite of the fact that in our Mtb SeMet-BfrA structure the propionate interactions cannot be analyzed, differences in the haem-binding pocket do exist when compared to other Bfr structures. For example, Arg53 in the haem-binding pocket of Mtb SeMet-BfrA has flipped orientation compared to that of Arg53 in *M. smegmatis* Bfr (Figure 5). This flipped conformation in our Mtb structure coincides with the formation of a salt bridge with Glu57, which is a threonine in the homologues from saprophytic mycobacterial species and may have physiological significance. This altered orientation may be responsible for juxtaposition of haem in a way so as to produce unusual γ-regioselectivity for the haem degradation reaction. It may be relevant here to note that no gene encoding HO (specific for cleavage at α-meso position of haem) has been identified so far in Mtb, which would further support this unusual regioselectivity. Fortuitous trapping of an atypical degraded product formed from its physiological substrate haem in a bacterioferritin structure may be indicative of a possible link between these iron storage proteins and haem catabolism/regulation under conditions currently not yet understood.

### Structural Consequences of Substituting Sulfur with Selenium: Demetallation of Haem

The electron density map in the haem-binding pocket of our SeMet-BfrA structure is well defined around the porphyrin ring but presents negative difference density at the position of the metal ion suggestive of absence of iron in the prosthetic group (Figure 4). The absorption spectra of SeMet-BfrA in solution are similar to the native Mtb BfrA absorption spectra (Figure 2a & 2b). These spectra were recorded for the protein samples before setting them up for crystallization, and demetallation/degradation process could have transpired during the period required for crystallization or during X-ray exposure. To eliminate the effect of X-radiation on haem demetallation and degradation, we performed single crystal microspectroscopy on native and SeMet-BfrA crystals before and after X-ray exposure (Figure 2c & 2d). These experiments clearly demonstrated that the X-ray beam from synchrotron radiation reduced the native BfrA crystal. However, absence of haem signatures near infra red region of the absorption spectra of SeMet-BfrA crystals (even with high dose of X-radiation) indicated that transformation of haem to demetallated/degraded porphyrin occurred during crystallization process.

The axial ligands coordinating haem iron in BfrA are furnished by thioether sulphur of Met52 from two symmetry related monomers. In our selenomethionyl analog, the sulphur to selenium replacement has resulted in changed electronic structure around haem moiety leading to demetallation of the haem molecule. Belief in this assumption gets strength from structure of native BfrA from Mtb (data not shown), where with natural Met52 as axial ligands to haem, the electron density associated with haem in this structure gives no indication of demetallation of the porphyrin IX, confirming this phenomenon to be specific to the selenium analog.

### The Ferroxidase Centre

The ferroxidase centre in the SeMet-BfrA structure has a low occupancy for both the Fe1 and the Fe2 sites. Low occupancies for irons have also been observed in the ferroxidase centres of *D. desulfuricans* and *R. capsulatus* Bfrs [7], [34] and were attributed to the possibility of iron leaving the di-iron site to the solvent through the pore or translocating into the inner core via a concerted movement of two residues that lie below the iron position, Glu47 and His130. Lack of resolution and sample heterogeneity (vis-à-vis iron occupancy) as the diffraction data was merged from multiple crystals, might be additional factors contributing to low occupancy of di-iron sites in our structure. A close examination of the di-iron site in our crystal structure reveals that this structure is compatible with the reduced form of bacterioferritin. The μ-oxo bridge present in the oxidized state is not seen in our structure and the di-iron centre appears to be photo-reduced by the synchrotron X-ray beam. Reported iron-iron distances in the oxidized di-iron centre are in between 3.1 and 3.4 Å. Upon reduction these distances are known to increase to over 3.8 Å resulting in a ligand rearrangement and loss of the μ-oxo bridge. In addition, for the reduced form of the protein, a conformational change has been observed for His130 (*A. vinelandii* Bfr; PDB entry: 1sof) and the average distance between Fe2 and the amino nitrogen ND1 of His130 is increased to 3.5 Å [35] as against average distance of 2.3 Å in the oxidized form (PDB entry: 2fl0) [36]. In our structure, His130 exhibits this alternative conformation, and side chain of Tyr25 residing in the vicinity of the di-iron binding site has moved away to make room for this conformation (Figure 6 inset). The movement of His130 seems to assists the migration of Fe2 towards the iron core. The distances between two metal ions reported in other Bfrs vary depending on the oxidation state of the metal. Average iron to iron distance in *D. desulfuricans* Bfr was found to be 3.71 Å in the ‘as isolated’ structure and 3.99 Å in the reduced structure [7]. In *M. smegmatis* Bfr the average distance between the two zinc ions have been reported to be 4.0 Å [19], whereas in our structure this distance between the two metal sites ranges from 4.0 to 4.6 Å (average of six subunits is 4.4 Å), indicating reduced metal status in both mycobacterial Bfrs. We do not consider these differences in the distance between the two metal ions in all reported structures to be significant as all of them (including ours) are at moderate resolution and hence, have certain uncertainties associated with the atomic positions. Overall, the ferroxidase centre is remarkably similar to that seen in other bacterioferritins [7], [19], [36], [37].

### Electron Funnel

**Figure 8:**
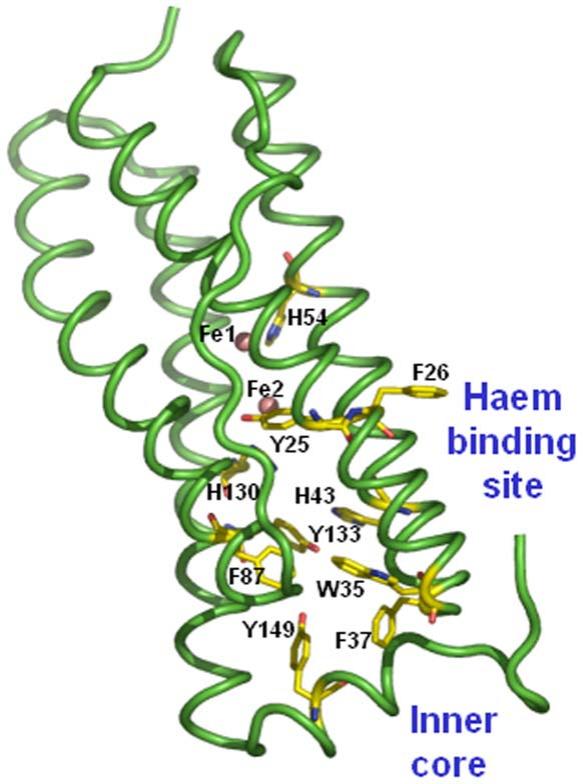
Predicted electron funnel between inner core and ferroxidase centre/haem binding site in Mtb SeMet-BfrA. Residues involved in forming the possible electron transfer path are shown as sticks (in atom type color). The metal ions of the di-iron centre are shown as brown spheres.

**Figure 9:**
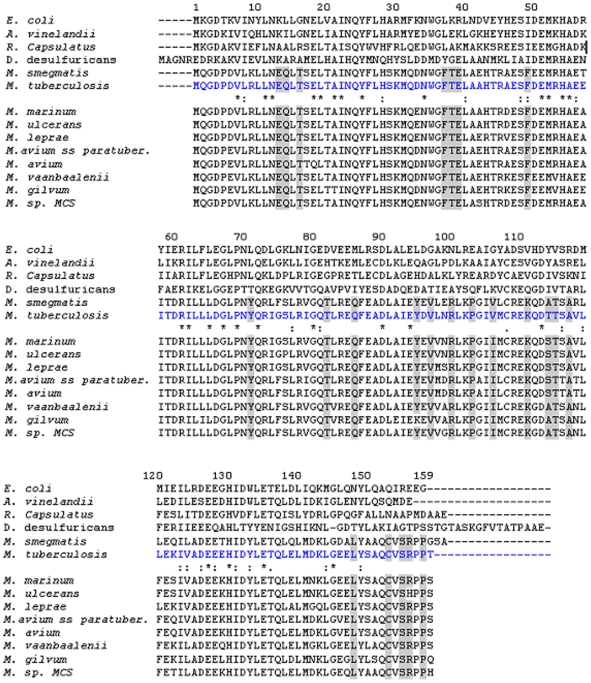
Amino acid sequence alignment of representative bacterioferritins. Sequences of Bfr representatives with known structures are placed above the Mtb BfrA sequence (colored blue) whereas annotated sequences from other mycobacterial species are placed below. The numbering of the residues is based on Mtb BfrA sequence. The alignment is underlined with the symbols *, :, . describing different degrees of sequence conservation in descending order. The boxes in gray represent conserved and unique residues among the mycobacterial species.

Studies on *E. coli* Bfr have demonstrated that as opposed to human ferritin, the ferroxidase centre in the bacterioferritins is stable in the oxidized form (as μ-oxo-bridged diferric centre) and does not spontaneously return to the apo form [21], [38]. Further, Baaghil et al. suggested that the electrons produced by the ongoing mineralization reaction in the bacterioferritin core are funneled back to the ferroxidase centre, where they reduce the μ-oxo-bridged diferric species to a diferrous centre [39]. According to the mechanism proposed by them, the growing iron oxide core is the site for overall Fe^2+^ oxidation, but reduction of oxygen occurs only at the ferroxidase centre. Hence, it is important to have a functional ferroxidase centre in Bfrs. In the presence of an oxidant but in absence of Fe^2+^, conditions known to express Mtb BfrA protein, the mineralization reaction cannot occur. Regeneration of the diferrous active site and recycling the iron would require an alternative source of electrons to reduce the μ-oxo-bridged diferric species back to the diferrous centre. In *D. desulfuricans* Bfr, an electron transfer path comprising a sequence of tyrosine and phenylalanine residues, positioned along the internal side of the four-helix bundle, has been proposed through which electrons travel to reduce the Fe^3+^ in the mineral lattice to Fe^2+^. In our Mtb SeMet-BfrA structure, a path with a slightly different distribution of residues (involving Tyr, Phe, Trp and His) accessible only through the di-iron centre and opening into internal cavity of the molecule can be located (Figure 8). However, the Phe residues (Phe63, Phe109, Phe125) and Tyr106 present in between the 3-fold channel and the di-iron site in *D. desulfuricans* Bfr are substituted by other non-aromatic amino acids in Mtb BfrA (Figure 9).

### Residues Conserved and Unique to Mycobacterial Species

**Figure 10:**
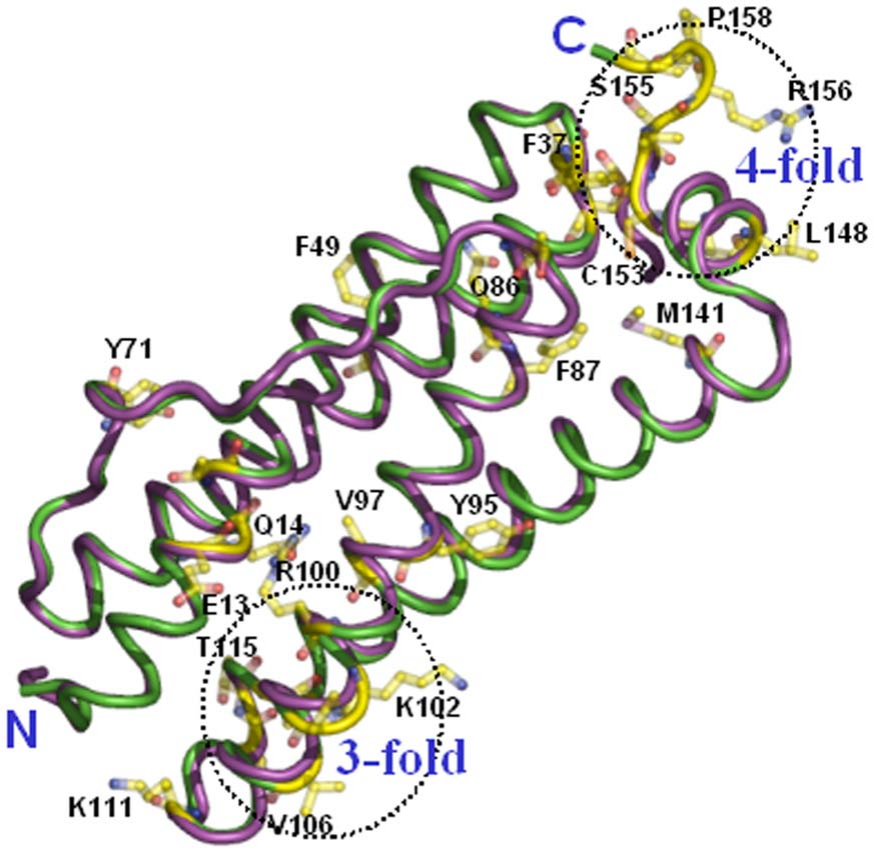
Unique regions specific to Mtb BfrA. Mapping of unique and conserved residues in mycobacteria. Residues unique to mycobacterial species (depicted by gray boxes in the sequence alignment figure 9) are labeled and depicted as sticks (in atom color) on cartoon representation of Mtb SeMet-BfrA monomer (green). The *E. coli* BfrA monomer (1bcf∶I; violet cartoon) is also superimposed to illustrate a dissimilar C-terminus.

In addition to the conserved residues of bacterioferritin superfamily (depicted by * in Figure 9), the multiple sequence alignment identifies additional conserved residues/regions specific to the *Mycobacterium* species (depicted by gray boxes in the sequence alignment figure 9). Interestingly, most of these residues seem to be clustered at the 3-fold and 4-fold junctions (Figure 10). The sequence Phe37Thr38Glu39 forms a turn between helices A and B and interacts with the unique extended C-terminus of Mtb SeMet-BfrA (154–159, Figure 10) which incidently in *E. coli* BfrA (shown in violet) loops in the other direction. This extended Mtb C-terminus interestingly has two consecutive prolines (Pro157 & Pro158). A proline substitution for the conserved Leu134 in the structure of H-ferritin has been shown to result in the localized unfolding at the 3-fold axis and an increase in the iron exit rates. Localized flexibility of ferritin at this junction, caused by substitution of proline for the conserved Leu134, was proposed to be akin to a “dynamic shutter” that could regulate iron release *in vivo* in response to cytoplasmic factors [40]. A similar shutter/gated mechanism for the C-terminus end might be envisaged in Mtb or alternatively, the two consecutive prolines placed at this position may have other function such as bridge the mineral core and Phe37 for facilitating electron transfer (Figure 10). Juxtaposition of Cys153 and Met141 amino acids (both unique but conserved in Mtb species), one on either side of Tyr149–a conserved residue in Bfr family (with one conservative substitution to Phe in *R. Capsulatus*) is very intriguing. Residues Tyr149 and Cys153 are part of E helix that provides stable interactions at the four-fold axis. In addition, these residues may also be involved in electron transfer between core metal and ferroxidase centre. Both Cys and Met amino acids can be oxidized to disulfides and methionine sulfoxide, respectively [41]. The reversible nature of these modifications may actually allow these amino acids to serve a protective role by detoxifying the local oxidative species [42] causing damage to conserved Tyr149. Tyr71 is placed in the long extended L-loop that from two subunits encloses the haem ligand and hence is expected to modulate haem-binding affinity of the protein.

Though for future understanding and resolving complexities associated with Mtb BfrA (at levels namely - iron entry, oxidation, translocation, mineralization and exit on one hand and functional role of haem on the other), further functional analysis with site specific Mtb BfrA mutants is essential, we believe targeting the unique C-terminus end of the protein should possibly result in specific inhibition of mycobacterial bacterioferritins.

### Role of Bacterioferritin in Mtb

It is quite evident that role of this protein family in biological system extends far beyond mere iron storage. It encompasses redox and non-redox catalysis, gene transcription regulation, sensing of iron and oxidative stress and DNA damage recognition and repair [10]. Inherent to this Mtb SeMet-BfrA supramolecular structure is the potential for localized responses to environmental perturbations as illustrated by our crystal structure. Here a local substitution of methionine with selenomethionine results in demetallation and nonenzymatic cleavage of haem, the latter known to occur generally in presence of ROS [32]. It appears that the haem ligand in Mtb SeMet-BfrA exists in an environment sensitive to ROS, which in the selenium substituted version results in ring opening and release of haem iron. It is remarkable how nature has devised a variety of different scaffolds and architectures to bind haem and modulate its function accordingly. The versatile function of haem relies on the delocalized π-electron system of the porphyrin ring. The redox properties of the central iron atom and the spectrum of interactions are provided by numerous variable protein scaffolds [43]. Mtb SeMet-BfrA structure displays a possible lost protein interaction with haem propionates and coincidentally Arg53-Glu57 salt bridge responsible for this loss appears to be unique to Mtb (Fig. 9), advocating a subtly altered haem-binding pocket in this organism. It is quite possible that selenium substitution in conjunction with an altered haem pocket in Mtb led to nonenzymatic cleavage of haem. Certainly this phenomenon has not been observed till date in haem bound to Bfrs from other organisms. If this is a physiologically relevant occurrence, our structure unfurls a not yet considered function of Mtb BfrA related to haem degradation.

Interestingly, the Mtb *bfr*A gene is produced under both low- and high-iron conditions, indicating an additional role for Mtb BfrA other than iron detoxification and storage [44], [45]. It is an attractive possibility that basal expression of BfrA is maintained under low iron state in Mtb not for iron storage but for release of iron, making it available for regulatory and synthetic purposes. If haemolysis in Mtb BfrA is a physiological event, it will naturally release the required free iron. Release of free iron may cause oxidation of local susceptible amino acids in the macromolecule leading to metal catalysed oxidation of the protein. It is now an established fact that protein oxidation is a physiological means by which proteins are “marked” for proteolytic degradation. Such a proteolytic pathway may further release iron and/or in parallel provide a valuable line of “secondary antioxidant defense” [46].

### Concluding Remarks

A static crystal cannot unveil the dynamic biology of protein as complex as bacterioferritin, that has evolved under strong structural constraints to accomplish functional needs which vary with the complexity of the organism. The basic function seems to be iron storage and detoxification when excess iron is present in the cell. But recent data have shown a large variability within this common theme. Our structure provides vital clues that may have remarkable implications on the possible function(s) of BfrA that have not yet been deliberated for this family of proteins. In addition, if the role of Mtb BfrA in the virulence of the organism is validated, the three-dimensional structure of this target provides a framework for rational structure-based designing of specific inhibitors.

It is usually implied that the replacement of methionine by selenomethionine in the protein structure does not induce any functional change in the protein. Our study nevertheless presents evidence against this general assumption. Though substitution of methionine by selenomethionine is considered to be a conservative replacement, differences do exist in the pK_a_ values and redox potentials of selenols and thiols [47]. Moreover, the van der Waals radius of selenium is slightly larger than that of sulphur and selenolate has greater electron donating capacity as compared to thiolate. This more electron-donating haem axial ligand in SeMet-BfrA facilitates the oxidation of its natural substrate as well as the removal of coordinated iron metal from the porphyrin nucleus. Whatever the mechanism for haem demetallation and degradation in our selenium-substituted structure, this study also provides a unique methodology to probe the role of axial ligands to haem in bacterioferritin protein family. The replacement of the natural ligand methionine with selenomethionine modifies its nucleophilic, radical and redox character. Insights acquired from the modified chemistry/environment of haem may further offer leads to already ongoing efforts to decipher the role of this molecule on bacterioferritin chemistry.

## Methods

### Expression and Purification of Selenomethionyl Analog of *M. tuberculosis* Bacterioferritin A (SeMet-BfrA)

*E. coli* BL21 (DE3) cells were transformed with recombinant plasmid pET21c-BfrA [17] harboring *M. tuberculosis bfrA* gene (Rv1876). A single transformed colony was inoculated in 5 ml LB medium and grown for 6 hours at 37°C. 500 ml LB (supplemented with 100 mg/L ampicillin) was inoculated with this 5 ml primary culture and grown for 12 hours at 37°C. The overnight culture was centrifuged at 5000 rpm for 10 minutes and the pellet was resuspended in 500 ml M9 minimal medium supplemented with 0.4% glucose, 2 µg/ml thiamine, 2 mM MgSO_4,_ 50 mg/L ampicillin, 100 µg/ml each of Lysine, Phenylalanine and Threonine and 50 µg/ml each of Isoleucine, Leucine, Valine and L-Selenomethionine. Cells were incubated for 30 minutes at 37°C and induced with 1 mM IPTG for 16 hours at 37°C followed by centrifugation at 6,000 rpm for 10 minutes at 4°C. The cells were resuspended in 20 ml of buffer (20 mM Tris pH 8.0, 50 mM NaCl, 1 mM PMSF and 2 mM β- mercaptoethanol) and lysed by using French press. The resulting lysate was centrifuged at 15,000 rpm for 30 minutes at 4°C and supernatant was collected.

Our earlier reported purification protocol had to be modified, as the final yield of native protein purified with Strep-Tactin column was very low (∼0.5 to 1 mg/L of culture). Therefore, recombinant SeMet-BfrA protein in the lysate was precipitated by adding ammonium sulfate (25–50% saturation) followed by stirring for 2 hours at 4°C. The precipitated protein was collected by centrifugation at 15000 rpm for 30 minutes at 4°C and resuspended in 5 ml of buffer (20 mM Tris pH 8.0, 50 mM NaCl, 1 mM PMSF and 1 mM DTT). The solubilized protein was loaded on pre equilibrated Sephacryl-300 gel filtration column and 5 ml fractions were collected. Fractions corresponding to expected molecular weight were pooled after analyzing for purity on SDS- PAGE, concentrated to 7.7 mg/ml and stored at −70°C.

### UV-Visible Spectroscopy

UV-visible spectra of the purified native BfrA and SeMet-BfrA proteins were recorded on a Shimadzu UV 1700 spectrophotometer in 1-cm path length cuvettes. Absorption maxima of the reduced proteins were obtained after addition of a few crystals of sodium dithionite directly to the sample in the cuvette.

### Crystallization of SeMet-BfrA

SeMet-BfrA crystallization was attempted under the same conditions as described earlier for the native protein [17]. Reddish brown crystals with similar morphology and size as the native BfrA crystals appeared within a few days.

### Data Collection and Processing

Diffraction of SeMet-BfrA crystals was checked with synchrotron radiation source of X11 beamline (European Molecular Biology Laboratory, Hamburg, Germany). Initially, to collect cryo-cooled data many cryoprotectants such as glycerol, dextrose, oils were tried and though crystals could be cryoprotected, the process resulted in diffused pattern and lowering of the diffraction resolution. Finally, crystals were mounted in quartz capillaries for room temperature data collection. In the initial diffraction images spots extended beyond 2.0 Å but unfortunately the high resolution spots died off quickly due to severe radiation damage and eventually after 20–25 frames crystals stopped diffracting completely. Despite forming crystals under similar solution conditions and having similar cell dimensions as compared to the native BfrA crystals, the space group of SeMet-BfrA appeared to be I4 instead of P4_2_. To maximize the completeness of the diffraction data many crystals were screened. Crystals were not perfectly isomorphous and only 4 crystals contributed to the final dataset. Also the resolution limits for different crystals were heterogeneous and this significantly reduced the resolution of the final merged dataset. The data was integrated and scaled with AUTOMAR (http://www.marresearch.com/automar/automar/run.htm) in steps to eliminate the bad crystals and images that showed severe radiation damage. First, the images with significant decrease in resolution were identified by visual inspection and were eliminated. Then reflection files from each crystal were independently scaled, to exclude non-isomorphous and ‘bad’ crystals having higher *R*_merge_ from further processing. Though the relative intensities of integrated images were scaled, data sets could not be merged with acceptable R-factors. The space group I4 can be indexed in two mutually non-compatible ways and therefore plausible rotation matrix was applied on the data sets before scaling and merging them. Considering the weak average intensities of the data sets, the R_merge_ of ∼10% is very acceptable. Data collection and processing statistics for SeMet-BfrA crystals are summarized in Table 2.

**Table 2:** Data collection and refinement statistics for Mtb SeMet-BfrA crystals.

Statistics	SeMet-BfrA
Diffraction data	
Space group	I4
Unit-cell parameters (Å)	a = 125.96,b = 125.96,c = 175.84
Temperature (K)	295
Wavelength (Å)	0.8148
Crystal-to detector distance (mm)	210
Resolution limit (Å)	25−2.5 (2.59−2.50)
Exposure time per image (s)	60
No. of observed reflections	205530
No. of Unique reflections	45901
Completeness (%)	99.4 (97.1)
Average redundancy	4.4 (4.1)
Mean *I/σ(I)*	6.7 (1.6)
† *R*merge (%)	10.3 (29.4)
No. of molecules in ASU	6
Matthews coefficient (Å3 Da-1)	3.06
Solvent content (%)	59.8
Refinement and model quality	
‡Rwork/§Rfree (%)	19/23
rms deviation bond lengths (Å)	0.005
rms deviation bond angles (°)	0.751
Average B factor (Å^2^)	31.4

Values in parentheses are for the highest resolution shell.

† *R*_merge_ = ∑*_hkl_*∑*_i_* |*I_hkl_*−<*I_hkl_*>|/∑*_hkl_*∑*_i_I_hkl_*, where *I_hkl_* is the intensity of an individual measurement of the reflection with Miller indices *h*, *k* and *l* and <*I_hkl_*> is the mean intensity of redundant measurements of that reflection.

‡ *R*_work_ = Σ*_hkl_* |*Fo_(hkl)_*−*Fc_(hkl)_*|/Σ*_hkl_* |*Fo_(hkl)_*|, where *Fo* and *Fc* are observed and calculated structure factors, respectively.

§ *R*_free_ calculated for a randomly selected subset of reflections (5%) that were omitted during the refinement.

### Structure Solution and Refinement

Molecular replacement was attempted with the PHENIX AutoMR Wizard [48], [49]. The Matthews parameter calculations [50] established that the asymmetric unit of SeMet-BfrA crystal contained 6 monomers. The homologous bacterioferritin structure from *M. smegmatis* (87% sequence identity) has 12 monomers in the asymmetric unit [19]. The search model used for molecular replacement was half of *M. smegmatis* Bfr 12-mer (PDB: 3bkn). The MR calculations gave a single and clear solution. Rebuilding of the protein chain to take into account the differences in sequence and chain length between *M. smegmatis* Bfr and Mtb SeMet-BfrA was carried out with the PHENIX AutoBuild Wizard. The structure obtained was further refined with the PHENIX refinement package phenix.refine [51] with iterative manual model building in COOT followed by density modification and refinement with phenix.refine. Water molecules were positioned into well-defined positive (*F_o_−F_c_*) difference densities with a lower cutoff of 3σ, if they participated in hydrogen bonds to either the protein or to well established water molecules. They were removed if their isotropic temperature B-factor refined to a value exceeding 60 Å^2^. Metal ions were placed into higher (*F_o_−F_c_*) difference densities based on B-values and nature of coordinating protein residues. In the final map some continuous electron density is visible beyond Thr159 in some subunits. Attempt was made to build the 2-residue linker (Ser-Ala) and sequence of Strep-tag at C terminus but only Ser-Ala residues could be build and were numbered 160–161. The B values of the Strep-tag residues were very high and hence not included in the final PDB. The quality of the final model was assessed with PROCHECK.

### Single-Crystal Microspectrophotometry

Absorption spectra of single crystal mounted in cryo-stream operating at ∼100 K were recorded using the microspectrophotometer installed on the X13 beamline synchrotron radiation source (EMBL, Hamburg). The crystal orientation was kept same for the measurements performed before as well as after the X-ray exposure to the crystal.

### Coordinates

Coordinates and structure factors for SeMet-BfrA are deposited in the RCSB Protein Data Bank (www.rcsb.org) under accession code 2wtl.

## Supporting Information

Figure S1 Mass spectrometry data of Mtb native and SeMet BfrA crystals. MALDI-TOF-MS analysis shows the presence of 616 Da haem peak in the Mtb native BfrA crystals (top panel) and absence of this peak in the SeMet-BfrA crystals (bottom panel).
(4.48 MB TIF)

## References

[1] O'Regan A, Joyce-Brady M (2001). Latent tuberculosis may persist for over 40 years.. BMJ.

[2] Stewart GR, Robertson BD, Young DB (2003). Tuberculosis: a problem with persistence.. Nat Rev Microbiol.

[3] Corbett EL, Watt CJ, Walker N, Maher D, Williams BG (2003). The growing burden of tuberculosis: global trends and interactions with the HIV epidemic.. Arch Intern Med.

[4] De Voss JJ, Rutter K, Schroeder BG, Barry CE (1999). Iron acquisition and metabolism by mycobacteria.. J Bacteriol.

[5] Rodriguez GM, Smith I (2003). Mechanisms of iron regulation in mycobacteria: role in physiology and virulence.. Mol Microbiol.

[6] Crichton R (2001). Inorganic Biochemistry of Iron Metabolism: From Molecular Mechanisms to Clinical Consequences..

[7] Macedo S, Romao CV, Mitchell E, Matias PM, Liu MY (2003). The nature of the di-iron site in the bacterioferritin from *Desulfovibrio desulfuricans*.. Nat Struct Biol.

[8] Andrews SC, Le Brun NE, Barynin V, Thomson AJ, Moore GR (1995). Site-directed replacement of the coaxial heme ligands of bacterioferritin generates heme-free variants.. J Biol Chem.

[9] Keren N, Aurora R, Pakrasi HB (2004). Critical roles of bacterioferritins in iron storage and proliferation of cyanobacteria.. Plant Physiol.

[10] Smith JL (2004). The physiological role of ferritin-like compounds in bacteria.. Crit Rev Microbiol.

[11] Cole ST, Brosch R, Parkhill J, Garnier T, Churcher C (1998). Deciphering the biology of *Mycobacterium tuberculosis* from the complete genome sequence.. Nature.

[12] Andrews SC (1998). Iron storage in bacteria.. Adv Microb Physiol.

[13] Miller CD, Kim YC, Walsh MK, Anderson AJ (2000). Characterization and expression of the *Pseudomonas putida* bacterioferritin alpha subunit gene.. Gene.

[14] Rachman H, Strong M, Schaible U, Schuchhardt J, Hagens K (2006). *Mycobacterium tuberculosis* gene expression profiling within the context of protein networks.. Microbes Infect.

[15] Gold B, Rodriguez GM, Marras SA, Pentecost M, Smith I (2001). The *Mycobacterium tuberculosis* IdeR is a dual functional regulator that controls transcription of genes involved in iron acquisition, iron storage and survival in macrophages.. Mol Microbiol.

[16] Bernard AR, Wells TN, Cleasby A, Borlat F, Payton MA (1995). Selenomethionine labelling of phosphomannose isomerase changes its kinetic properties.. Eur J Biochem.

[17] Gupta V, Gupta RK, Khare G, Salunke DM, Tyagi AK (2008). Cloning, expression, purification, crystallization and preliminary X-ray crystallographic analysis of bacterioferritin A from *Mycobacterium tuberculosis*.. Acta Crystallogr Sect F Struct Biol Cryst Commun.

[18] Wallace CJ, Clark-Lewis I (1992). Functional role of heme ligation in cytochrome c. Effects of replacement of methionine 80 with natural and non-natural residues by semisynthesis.. J Biol Chem.

[19] Janowski R, Auerbach-Nevo T, Weiss MS (2008). Bacterioferritin from *Mycobacterium smegmatis* contains zinc in its di-nuclear site.. Protein Sci.

[20] Laskowski RA, MM, Moss DS, Thornton JM (1993). PROCHECK: a programme to check the stereochemical quality of protein structures.. J Appl Crystallog.

[21] Le Brun NE, Andrews SC, Guest JR, Harrison PM, Moore GR (1995). Identification of the ferroxidase centre of *Escherichia coli* bacterioferritin.. Biochem J.

[22] Pereira AS, Small W, Krebs C, Tavares P, Edmondson DE (1998). Direct spectroscopic and kinetic evidence for the involvement of a peroxodiferric intermediate during the ferroxidase reaction in fast ferritin mineralization.. Biochemistry.

[23] Powell AK (1998). Ferritin. Its mineralization.. Met Ions Biol Syst.

[24] Douglas T, Ripoll DR (1998). Calculated electrostatic gradients in recombinant human H-chain ferritin.. Protein Sci.

[25] Ponka P (1999). Cell biology of heme.. Am J Med Sci.

[26] Ishii DN, Maniatis GM (1978). Haemin promotes rapid neurite outgrowth in cultured mouse neuroblastoma cells.. Nature.

[27] Granick JL, Sassa S (1978). Hemin control of heme biosynthesis in mouse Friend virus-transformed erythroleukemia cells in culture.. J Biol Chem.

[28] Abraham NG (1991). Molecular regulation–biological role of heme in hematopoiesis.. Blood Rev.

[29] Ryter SW, Tyrrell RM (2000). The heme synthesis and degradation pathways: role in oxidant sensitivity. Heme oxygenase has both pro- and antioxidant properties.. Free Radic Biol Med.

[30] Vercellotti GM, Balla G, Balla J, Nath K, Eaton JW (1994). Heme and the vasculature: an oxidative hazard that induces antioxidant defenses in the endothelium.. Artif Cells Blood Substit Immobil Biotechnol.

[31] Mancuso C, Barone E (2009). The Heme Oxygenase/Biliverdin Reductase Pathway in Drug Research and Development.. Curr Drug Metab.

[32] Bonnett R, McDonagh AF (1973). The meso-reactivity of porphyrins and related compounds. VI. Oxidative cleavage of the haem system. The four isomeric biliverdins of the IX series.. J Chem Soc [Perkin 1].

[33] Wang J, Evans JP, Ogura H, La Mar GN, Ortiz de Montellano PR (2006). Alteration of the regiospecificity of human heme oxygenase-1 by unseating of the heme but not disruption of the distal hydrogen bonding network.. Biochemistry.

[34] Cobessi D, Huang LS, Ban M, Pon NG, Daldal F (2002). The 2.6 A resolution structure of *Rhodobacter capsulatus* bacterioferritin with metal-free dinuclear site and heme iron in a crystallographic ‘special position’.. Acta Crystallogr D Biol Crystallogr.

[35] Liu HL, Zhou HN, Xing WM, Zhao JF, Li SX (2004). 2.6 A resolution crystal structure of the bacterioferritin from *Azotobacter vinelandii*.. FEBS Lett.

[36] Swartz L, Kuchinskas M, Li H, Poulos TL, Lanzilotta WN (2006). Redox-dependent structural changes in the *Azotobacter vinelandii* bacterioferritin: new insights into the ferroxidase and iron transport mechanism.. Biochemistry.

[37] Frolow F, Kalb AJ, Yariv J (1994). Structure of a unique twofold symmetric haem-binding site.. Nat Struct Biol.

[38] Yang X, Le Brun NE, Thomson AJ, Moore GR, Chasteen ND (2000). The iron oxidation and hydrolysis chemistry of *Escherichia coli* bacterioferritin.. Biochemistry.

[39] Baaghil S, Lewin A, Moore GR, Le Brun NE (2003). Core formation in *Escherichia coli* bacterioferritin requires a functional ferroxidase center.. Biochemistry.

[40] Takagi H, Shi D, Ha Y, Allewell NM, Theil EC (1998). Localized unfolding at the junction of three ferritin subunits. A mechanism for iron release?. J Biol Chem.

[41] Stadtman ER (2006). Protein oxidation and aging.. Free Radic Res.

[42] Levine RL, Moskovitz J, Stadtman ER (2000). Oxidation of methionine in proteins: roles in antioxidant defense and cellular regulation.. IUBMB Life.

[43] Paoli M, Marles-Wright J, Smith A (2002). Structure-function relationships in heme-proteins.. DNA Cell Biol.

[44] Chen CY, Morse SA (1999). *Neisseria gonorrhoeae* bacterioferritin: structural heterogeneity, involvement in iron storage and protection against oxidative stress.. Microbiology.

[45] Carrondo MA (2003). Ferritins, iron uptake and storage from the bacterioferritin viewpoint.. EMBO J.

[46] Davies KJ (1986). Intracellular proteolytic systems may function as secondary antioxidant defenses: an hypothesis.. J Free Radic Biol Med.

[47] Patai S, Z R (1986). The Chemistry of Organic Selenium and Tellurium Compounds..

[48] Adams PD, Gopal K, Grosse-Kunstleve RW, Hung LW, Ioerger TR (2004). Recent developments in the PHENIX software for automated crystallographic structure determination.. J Synchrotron Radiat.

[49] Adams PD, Grosse-Kunstleve RW, Hung LW, Ioerger TR, McCoy AJ (2002). PHENIX: building new software for automated crystallographic structure determination.. Acta Crystallogr D Biol Crystallogr.

[50] Matthews BW (1968). Solvent content of protein crystals.. J Mol Biol.

[51] Afonine PV, Grosse-Kunstleve RW, Adams PD, Lunin VY, Urzhumtsev A (2007). On macromolecular refinement at subatomic resolution with interatomic scatterers.. Acta Crystallogr D Biol Crystallogr.

