# Supplementary figures and images for: Crystal Structure of Bfr A from Mycobacterium tuberculosis: Incorporation of Selenomethionine Results in Cleavage and Demetallation of Haem
Source: PLoS One. 2009 Nov 25;4(11):e8028. doi: 10.1371/journal.pone.0008028 (PMC2777505; doi:10.1371/journal.pone.0008028)

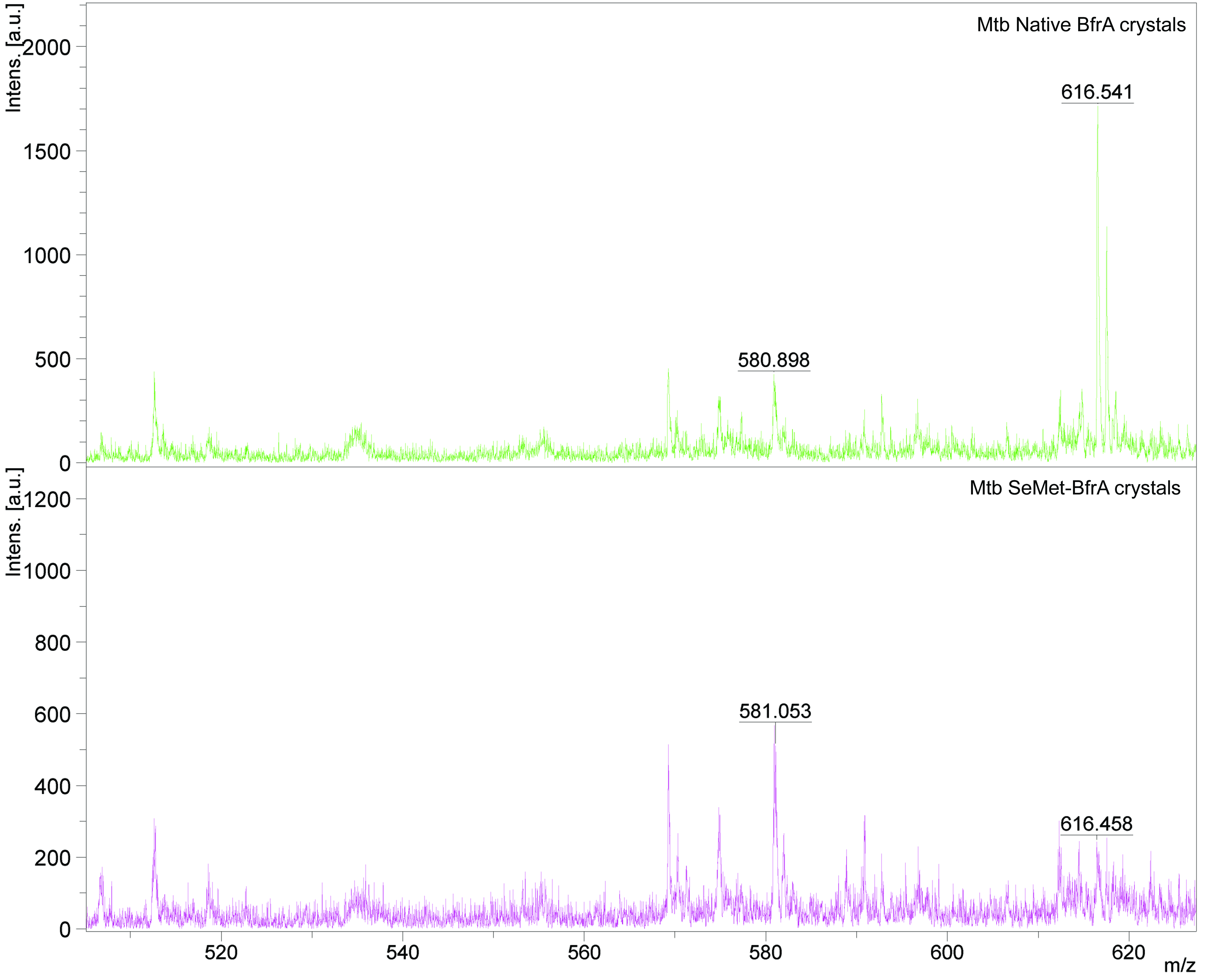

Supplement: Figure S1 — Mass spectrometry data of Mtb native and SeMet BfrA crystals. MALDI-TOF-MS analysis shows the presence of 616 Da haem peak in the Mtb native BfrA crystals (top panel) and absence of this peak in the SeMet-BfrA crystals (bottom panel). (4.48 MB TIF) [file pone.0008028.s001.tif]
